# Evaluation of Left Ventricular Function Long Term After Arterial Switch Operation for Transposition of the Great Arteries

**DOI:** 10.1007/s00246-018-1977-6

**Published:** 2018-09-10

**Authors:** S. W. van Wijk, M. M. P. Driessen, F. J. Meijboom, T. Takken, P. A. Doevendans, J. M. Breur

**Affiliations:** 1 Paediatric Cardiology, Wilhelmina Children’s Hospital, University Medical Centre Utrecht, Lundlaan 6, 3584 EA Utrecht, The Netherlands; 2 Cardiology, University Medical Centre Utrecht, Heidelberglaan 100, 3584 CX Utrecht, The Netherlands; 3 Child Development & Exercise Center, Wilhelmina Children’s Hospital, University Medical Centre Utrecht, Lundlaan 6, 3584 EA Utrecht, The Netherlands; 4 ICIN-Netherlands Heart Institute, Moreelsepark 1, 3511 EP Utrecht, The Netherlands

**Keywords:** Arterial switch operation, Transposition of the great arteries, Deformation imaging, Strain imaging, Left ventricular ejection fraction, Echocardiography

## Abstract

Long-term after arterial switch operation for transposition of the great arteries, abnormal coronary anatomy and altered loading conditions could compromise ventricular function. The current study investigates whether left ventricular function, measured with echocardiographic bi-plane ejection fraction and deformation imaging, in patients long term after arterial switch operation for transposition of the great arteries differs from healthy peers. A cross-sectional cohort study of patients at least 12 years after arterial switch operation was analyzed with bi-plane Simpson’s left ventricular ejection fraction (LVEF) and deformation (speckle tracking) echocardiography. 81 patients, median age 20.6 (interquartile range 13.5–28.4) years, were included. LVEF was normal on average at 55.5 ± 6.1%. Global longitudinal strain (GLS) was lower in patients compared to healthy peers throughout all age groups and on pooled average (− 15.4 ± 1.1% vs. − 23.2 ± 0.9%). Although LVEF is normal on average in patients after arterial switch operation for transposition of the great arteries, GLS is impaired compared to healthy peers. The reduced GLS could indicate sub-clinical myocardial dysfunction.

## Introduction

The arterial switch operation is the standard procedure for correcting transposition of the great arteries [1]. Current perioperative mortality is under 4% and long-term survival of perioperative survivors is around 97% at 25 years follow-up [2, 3].

However, long-term morbidity after arterial switch operation for correcting transposition of the great arteries is substantial and between 25 and 30% of patients require reinterventions [2, 4]. Also, compromise of ventricular function is feared on theoretical grounds. Firstly, sub-optimal coronary perfusion could lead to chronic ischemia inducing left ventricular (LV) damage and remodeling. The coronary arteries are reimplanted in a non-physiologic, sub-optimal position in the coronary sinuses, but higher in the aortic root and at a more acute angle [5]. Doubts exist whether coronary function (coronary flow reserve) is affected by this abnormal anatomy [6, 7].

Secondly, altered loading conditions could cause LV dysfunction. Aortic regurgitation is often present in adult patients due to aortic root dilatation, leading to increased preload. The presence of a circular vascular anastomosis a few centimeters distal of the aortic valve, and the acute angle of the aortic arch (also known as a gothic arch [8]) that develops in a majority of patients could cause increased afterload. These considerations, together with the knowledge that eventually a considerable number of adult patients with congenital heart disease will die from ventricular failure [9], make it likely that LV dysfunction will develop in a substantial portion of this specific patient population.

In line with these considerations, several studies have already been published assessing LV function. In most of these studies however, left ventricular ejection fraction (LVEF) was used as marker of LV systolic function. LVEF is normal in a large majority of patients long term after arterial switch operation, with about 10% of patients showing impaired ejection fraction [10–12]. An important limitation in most of these studies is the use of M-mode-derived LVEF (Teicholdz), which is an inaccurate method to assess LVEF.

Deformation imaging may be a more sensitive tool to detect early ventricular dysfunction. It has shown to accurately predict a decline in LV function in a variety of conditions, including heart failure in cardio-oncology [13]. This technique’s value is also proven in patients with congenital heart disease [14]. Limited data are published on deformation imaging of the LV in patients after arterial switch operation for transposition of the great arteries. Pettersen et al. [15] and Di Salvo et al. [16] reported abnormal global longitudinal strain (GLS) in pediatric patients, but data on deformation imaging in adults after arterial switch operation have not been reported. This is of particular interest since cardiovascular complications become increasingly likely to occur after childhood.

This article determines whether LV function long term after arterial switch operation for transposition of the great arteries differs from healthy peers, when measured with bi-plane LVEF or speckle tracking deformation imaging. We hypothesized that global deformation, as an early indicator of dysfunction, is decreased in a substantial portion of the arterial switch operation population, while this dysfunction goes unnoticed when measured by classical volume assessment.

## Methods

### Population

A cross-sectional cohort study was performed between August 2011 and January 2015. Patients eligible for inclusion had received an arterial switch operation for simple or complex dextro-transposition of the great arteries in our center and were over 12 years of age. Patients were examined by echocardiography and cardiopulmonary exercise testing within 1 day. The institutional review committee of the University Medical Center Utrecht approved this study. Informed consent was obtained from all patients and additional consent by parents if aged < 18 years of age. Patient characteristics were obtained from the patient chart.

Reference values for healthy controls were obtained from the study of Alcidi et al. [17] The paper by Alcidi et al. reports the closest match covering adolescents and young adults (both age groups covered in this study) with one consistent method of deformation ultrasound imaging in a comparable European population.

### Echocardiography

Doppler transthoracic echocardiography was performed on a Toshiba Artida (Toshiba, Tokyo, Japan) with a 5 MHz transducer. The peak velocity (continuous wave) was used as an index of valve obstruction. Valve regurgitation, *E*/*e*′, and LV mass were assessed according to guidelines [18] and LVEF was determined by bi-plane Simpson’s method. Left atrial area was assessed through manual trace in the four-chamber view. For strain measurements, three full cardiac cycles in four-chamber view were captured at > 50 Hz. Septal dyskinesia was defined as abnormal septal motion, not in phase with movement of the LV posterior wall.

Longitudinal strain analysis was performed with TomTec Image Suite 6.0 (TomTec Imaging Systems GmbH, Unterschleissheim, Germany). After assessment of full view of all parts of LV myocardium for at least one cycle, a six-segment analysis of endocardial longitudinal strain was performed. Segments were excluded if visual assessment of the analysis confirmed non-capturing by the software. If two or more segments showed non-capturing, the patient was excluded from analysis. End systolic and end diastolic frames were determined manually. Peak GLS was calculated automatically and reported in percent (%). Inter- and intra-observer reproducibility was checked through blinded reanalysis of random selected sample of 20 patients and tested with intraclass correlation coefficients.

### Cardiopulmonary Exercise Testing

To correlate between GLS, LVEF, and functional parameters, patients performed a cardiopulmonary exercise test using an electronically braked cycle ergometer (Lode Corival, Lode BV, Groningen, The Netherlands) on the same day as echocardiography. Before exercise, forced expiratory volume in the first second was measured. After a rest period of 3 min and 3 min of unloaded cycling, the work rate was increased in a ramp-like protocol with 15, 20, or 25 W/min. It was aimed to complete the test within 8–12 min. Maximum effort was defined as a peak respiratory exchange ratio of > 1.0 for children and > 1.1 for adults. Indications for premature test termination are provided by the American Thoracic Society/American College of Chest Physicians [19].

During exercise, oxygen uptake (VO_2_), carbon dioxide elimination (VCO_2_), and minute ventilation (VE) were recorded using a computerized breath-by-breath analyzer (ZAN 600, ZAN Meβgeräte GmbH, Accuramed BVBA, Herk-de-Stad, Belgium). Peak oxygen uptake (VO_2_peak%) was expressed as percentage of the predicted value, based on age, sex, height, and body weight. Ventilatory efficiency [minute ventilation relative to carbon dioxide elimination (VE/VCO_2_ slope)] was calculated using linear regression, up to the respiratory compensation point. Peak heart rate 85% or less, VO_2_peak 84% or less, and VE/VCO_2_ slope 34 or greater were considered abnormal.

### Statistical Analysis

Normality of distribution was checked using the Shapiro–Wilk test. Normally distributed continuous data were reported as mean ± standard deviation. Alternatively distributed data were expressed as median (interquartile range). Missing data were accounted for multiple imputations [20].

For comparison of groups, independent samples *t* test or ANOVA was used as appropriate. Average deformation across age groups was calculated through a pooled meta-analysis (fixed effect). Associations were tested with Pearson’s (continuous variables) or Spearman’s (categorical variables) correlation as appropriate. In multivariate analysis, entry value for variables was 0.05 and removal value was 0.1. Inter- and intra-observer reproducibility was expressed as intraclass correlation. For all tests, a *p* value of < 0.05 was accepted as statistically significant.

## Results

A total of 96 patients were approached for this study. In total, 81 participated in this study. Baseline characteristics are summarized in Table 1. The median age of participants was 20.6 years (interquartile range 13.5–28.4 years). The average LVEF was 55.5 ± 6.1% (Table 2). Eight patients had an impaired LVEF (< 50%), but all had an LVEF > 40%. Four of these patients had a complex TGA with VSD. Three patients showed septal dyskinesia on echocardiography. One of them had a Ross procedure followed by a mechanical aortic prosthesis and one had arrhythmia causing cardiac arrest post-operatively, though no signs of ischemia were found. The other three have no known cause for the impairment. None of these three patients showed signs of frank ischemia during surgery, post-operatively, or during exercise testing at follow-up. All patients functioned at NYHA class I. One patient had reconstruction of the left coronary artery origo. Other patients showed no signs of coronary dysfunction.

**Table 1:** Patient characteristics

Characteristic	Patients (*N* = 81)
Demographics	
Age, years (IQR)	20.6 (13.5–28.4)
Sex, male *N* (%)	59 (73)
Height (cm)	172 ± 12.7
Weight (kg)	64.6 ± 17.5
Surgical details	
Rashkind, *N* (%)	50 (69)
Two-stage arterial switch operation, *N* (%)	9 (13)
Lecompte, *N* (%)	64 (79)
Usual coronary anatomy^a^, *N* (%)	58 (72)
Simple transposition of the great arteries, *N* (%)	52 (64)
Age at procedure, days (IQR)	8 (5–18)
Time since procedure, years (IQR)	20.6 (15.3–26.0)

^a^Following Leiden classification

**Table 2:** Echocardiographic and exercise testing outcomes

Echocardiography	
LVEF (%)	55.5 ± 6.1
GLS (%)	− 15.35 ± 1.1
LVEDD/BSA (cm/m^2^)	2.92 ± 0.44
LVmass/BSA (g/m^2^)	75.4 ± 19.3
*E*/*E*′ (%)	6.5 ± 1.5
Left atrial area (cm^2^)	17.1 ± 2.4
Moderate or severe AR *N* (%)	5 (6.2)
Exercise testing	
VO_2_peak/kg (L/min)	40.24 ± 12.3
VO_2_peak/kg (%pred)	88 ± 23
HRpeak (min)	180 ± 17
HRpeak (%pred)	96 ± 9

*LVEF* left ventricular ejection fraction, *GLS* global longitudinal strain, *LVEDD* left ventricular end diastolic diameter, *BSA* body surface area, *AR* aortic regurgitation, *VO*_*2*_*peak* maximum oxygen uptake, *HRpeak* maximum heart rate

GLS could be assessed in 71/81 patients (87.7%); results are shown in Table 3, excluding patients with inadequate imaging. Three of the excluded patients had a diminished LVEF. GLS was significantly lower compared to reference data across all age groups. Pooled average GLS for all age groups was − 15.35 ± 1.13% (95% CI − 13.13 to − 17.56) for patients and − 23.15 ± 0.90% (95% CI − 21.94 to − 24.91) for controls (*p* < 0.001). Group-wise analysis showed no statistically significant differences between LVEF or GLS across age groups (*p* = 0.156 and *p* = 0.076, respectively). Inter-observer ICC was 0.854 and intra-observer ICC was 0.938. No significant difference was found between GLS in patients with simple or complex TGA (*p* = 0.677). GLS between patients with diminished LVEF did also not differ significantly from the other patients (*p* = 0.463).

**Table 3:** LV strain compared to healthy controls

Age group (years)	LVEF, % (*N*)^a^	GLS arterial switch operation, % (*N*)^a^	GLS control, % (*N*)^a^	*p* value GLS
12–19	56.9 ± 6.6 (42)	− 17.53 ± 3.4 (37)	− 23.5 ± 1.3 (45)	< 0.0001
20–29	54.4 ± 5.5 (33)	− 17.29 ± 3.2 (29)	− 23.1 + 1.8 (45)	< 0.0001
30–39	56.0 ± 4.7 (6)	− 13.60 ± 1.5 (5)	− 22.6 ± 1.7 (45)	< 0.0001

*LVEF* left ventricular ejection fraction, *GLS* global longitudinal strain

^a^No statistically significant differences between age groups

### Correlation Analysis

Bivariate analysis showed no statistical significant correlation between demographic factors, surgical details (as specified in Table 1), or echocardiographic or exercise capacity parameters (as specified in Table 2) and decreased GLS values (Table 4). Imaging features showed a weak correlation between strain and LVEF (correlation coefficient 0.221, *p* = 0.048), and LV end diastolic dimensions normalized for BSA (correlation coefficient − 0.311, *p* = 0.011). Table 4 shows all correlation coefficients. No statistical significant correlation was observed between exercise testing parameters with LVEF or LV end diastolic diameter as dependent variable.

**Table 4:** Univariate correlations of demographics, surgical properties, imaging features, and exercise capacity with GLS

	Correlation with GLS (*r*)	*p* value
Gender	0.153^§^	0.172
Follow-up	− 0.107	0.341
Age at arterial switch operation	− 0.177	0.115
Rashkind	− 0.103^§^	0.361
Lecompte	0.055^§^	0.627
Pulmonary banding	− 0.140^§^	0.211
VSD	− 0.093^§^	0.411
Coronary pattern^a^	− 0.040^§^	0.724
LVEF	0.221	0.048
LVEDD	− 0.272	0.017
LVEDD/BSA	− 0.311	0.011
LVmass	− 0.083	0.478
*E*/*E*′	− 0.011	0.926
Left atrial area	0.096	0.396
AR	0.061^§^	0.232
VO_2_peak/kg (% pred)	0.065	0.577
HRpeak (%pred)	− 0.024	0.836
VE/VCO_2_ slope (%pred)	− 0.080	0.504

^a^1LCx, 2R considered normal, any other configuration considered variant

^§^Spearmans correlation coefficient, rest is Pearsons

Multiple correlation analysis showed no statistically significant correlation between any of the tested parameters with GLS as dependent variable.

## Discussion

Long term after arterial switch operation for transposition of the great arteries GLS is reduced in patients, across all age groups. In contrast, LVEF is normal on average in patients after arterial switch operation, with only 8 (9.9%) patients exhibiting a reduced LVEF. Although LVEF was normal on average, the reduced deformation imaging could indicate sub-clinical myocardial dysfunction.

In our study, the decrease in GLS could not be explained by surgical or anatomical factors. No correlation was found between GLS and demographics, surgical details, coronary anatomy, or presence of a VSD. This lack of correlation aligns with findings by Pettersen et al. [15]. The only factors that were weakly correlated to GLS were LV end diastolic diameter and LVEF and none remained in multiple regression analysis. Therefore, the mechanism through which deformation is decreased remains unclear.

Di Salvo et al. recently reported a decrease in longitudinal strain after arterial switch operation in younger patients (average age 8.7 years) [16]. They showed a significant correlation between older age at the time of arterial switch operation and lower GLS values on echocardiography. Our results do not reproduce this relationship after a median follow-up of 20.6 years. This could in part be explained by a different patient selection. Where Di Salvo et al. reported on a population with single-stage arterial switch operation only, our study’s cohort had 13% of patients who received a pulmonary banding followed by late arterial switch operation. Furthermore, Kramer et al. showed a significant difference in LVEF between one- and two-stage arterial switch patients [21]. In our data, subgroup analyses of these separate groups showed no difference in GLS or LVEF.

The decreased GLS in our cohort could mean it either decreases over time or it could be a pre-existing condition as a direct consequence of pre- and perioperative myocardial damage through ischemia. Klitsie et al. found that LV function (systolic and diastolic) recovers to values comparable to controls in the first year after arterial switch, after taking an initial hit [22]. Malakan Rad et al. reported normal strain in patients with simple transposition of the great arteries at a mean age of 15 months [23]. The results of Malakan Rad et al. indicate GLS could decline over time, although no significant correlation was found between age and strain in our data.

Several mechanisms could explain why deformation would decrease over time. One could be through mild ischemia from altered coronary blood flow. No patients showed signs of frank ischemia during exercise testing in our study. However, this does not exclude the possibility of subtle, sub-clinical ischemia in this patient group. Another cause could lie in altered loading conditions; patients are at risk for aortic root dilatation and regurgitation. Furthermore, a portion of patients after arterial switch operation develops a gothic aortic arch, which in itself could cause higher afterload and in turn predisposes for aortic root dilatation and regurgitation [24].

Deformation imaging has been validated in the field of cardio-oncology. In congenital heart disease, longitudinal follow-up on the predictive value of deformation imaging in young patients has yet to be performed. Therefore, clinical significance of decreased GLS is still undetermined. It is however, currently accepted as a more sensitive and robust marker of LV function than LVEF [25].

Even though long-term clinical outcome is considered excellent in patients after arterial switch operation for transposition of the great arteries for the duration of follow-up now available [2], the decrease in GLS gives rise to concern for long-term preservation of LV function. Nevertheless, in the age interval we studied in our cross-sectional cohort, there was no further decline in GLS with advancing age, although not statistically significant. Long-term longitudinal follow-up studies are needed to confirm if abnormal strain predisposes for worse clinical outcome in patients with transposition of the great arteries. Based on these results, strain analysis should be incorporated in longitudinal follow-up of patients with an arterial switch operation after transposition of the great arteries.

### Limitations

Our study was limited by the use of different speckle tracking software between our cohort and reference data. However, several studies showed good comparability in GLS between vendors [26, 27].

Furthermore, this study includes a heterogenic patient population of simple and complex TGA patients and one- and two-stage arterial switch patients to best represent long-term outcomes of the entire patient group long after the arterial switch operation. Subgroup analysis showed no significant differences between these groups. It also includes patients with aortic regurgitation, which negatively affects GLS. Again, these patients are included to provide the best representation of patients after the arterial switch operation, since aortic regurgitation is a common finding in these patients.

More comprehensive follow-up research is needed to look into the mechanism of reduced deformation. Our data showed no correlation with exercise parameters. However, altered loading conditions through abnormal aortic arch shapes and increased vascular stiffness were not directly included in this study.

## Conclusion

At long-term follow-up, GLS is impaired in patients after arterial switch operation for transposition of the great arteries. Whether the reduction in GLS precedes cardiovascular morbidity and mortality requires longer follow-up. LVEF is normal on average; select cases show mildly impaired function.
